# Sentinel lymph node mapping in post chemotherapy nonseminoma testicular cancer patients undergoing retroperitoneal lymph node dissection: A series of nine cases

**DOI:** 10.22038/AOJNMB.2021.55218.1380

**Published:** 2022

**Authors:** Leili Zarifmahmoudi, Hamidreza Ghorbani, Ramin Sadeghi, Kayvan Sadri, Salman Soltani, Atena Aghaee

**Affiliations:** 1 Nuclear Medicine Research Center, Mashhad University of Medical Sciences, Mashhad, Iran; 2 Kidney transplantation complications research center, Mashhad University of Medical Sciences, Mashhad, Iran

**Keywords:** Nonseminoma, Retroperitoneal lymph node dissection, Chemotherapy, Sentinel lymph node, Nuclear medicine, Cancer

## Abstract

**Objective(s):**

Testicular germ cell cancers are the most common solid malignancy among young men at the age ranging between 14 and 35 years. In this study, we evaluated the feasibility of sentinel lymph node mapping using intraoperative injection of radiotracer in nonseminomatous testicular cancer patients with history of orchiectomy who were candidate for retroperitoneal lymph node dissection (RPLND) in post-chemotherapy setting.

**Methods:**

Nine consecutive cases were included in the study. Technetium-99m-labelled phytate was injected in two divided doses in the stump of the spermatic cord, through transabdominal approach. A hand-held gamma probe was used for radio-guided retroperitoneal sentinel lymph node detection intraoperatively and confirming the location of the sentinel lymph nodes.

**Results:**

Detection rate and the false negative rate were estimated as the main indices. The detection rate was 6/9 (66%) and the false negative rate was 0/2 (0%). Location of the dissected sentinel lymph nodes were interaortocaval (2 patients), internal iliac (1 patient), external iliac (1 patient), common iliac (2 patients), and paraaortic (1 patient).

**Conclusion:**

Sentinel lymph node mapping technique seems to be feasible and promising in post chemotherapy non-seminoma testis cancer patients who are candidate for RPLND; however, further larger studies are needed to increase and standardize the detection rate.

## Introduction

 Testicular cancer is a rare tumor which accounts for almost 1-5% of malignancies in men. Testicular germ cell cancers are the most common solid malignancy among young men at the age ranging between 14 and 35 years (1). The incidence of testis cancer has shown an increasing trend over the last 40 years with substantial differences among countries (2). 

 Nonseminoma is the more aggressive type of testicular germ cell tumors (GCTs) compared with the seminoma type, which comprises multiple cell types of embryonal carcinoma, choriocarcinoma, yolk sac tumor, and teratoma (3, 4).

 The treatment options for nonseminomatous GCTs after the radical orchiectomy include surveillance, chemotherapy, and retroperitoneal lymph node dissection (RPLND), which will be decided following retroperitoneal and chest evaluations and repeated measurements of beta human chorionic gonadotropin (beta-hCG) and alpha-fetoprotein (AFP) serum tumor markers (5, 6). RPLND is usually the standard of care and first line therapy for post-chemotherapy non-seminoma patients with negative or plateau tumor marker levels and abnormal imaging findings (residual masses ≥ 1 cm) (7, 8). In these patients, there is a risk of existing mature teratoma and/or viable GCT, which indicates the necessity of post-chemotherapy RPLND to determine the extent of the spread of any malignant disease and to resect all the residual masses in the retroperitoneum. RPLND is a challenging surgery that should be done in high-volume centers and might be accompanied with adjunctive surgeries including nephrectomy and vascular surgery (9, 10). Complication rate of 7% -30% has been reported for post-chemotherapy RPLND including retrograde ejaculation, serious bleeding, and lymphatic leak (11, 12). Finding a less aggressive staging technique will reduce the morbidity and mortality rates accompanied with RPLND and might be able to avoid the unnecessary RPLNDs.

 Introduction of sentinel lymph node biopsy as a less aggressive approach to lymph node dissection has replaced regional lymphatic evaluation in melanoma, breast, gynecological and urological malignancies (13-19). Sentinel lymph node as the first metastatic tumor landing site through lymphatic flow, can be detected using radiotracers or dyes (20, 21). The dissected sentinel lymph node reveals the pathologic status of other regional lymph nodes and there is a potential benefit of avoiding unnecessary lymph node dissection.

 Sentinel node mapping has also been evaluated in urological oncology. Although the feasibility and accuracy of sentinel lymph node mapping has actively been evaluated in prostate, penile, bladder, and renal cancers, few studies have considered the validity of this staging approach in testicular cancer patients at the time of orchiectomy (12, 15, 19, 22, 23).

 In this study, we evaluated the feasibility of sentinel lymph node mapping using intra-operative injection of radiotracer in non-seminomatous testicular cancer patients with history of orchiectomy who were candidate for RPLND in post-chemotherapy setting.

## Methods

 In this pilot study, all patients with non-seminoma germ cell tumors who were candidate for post-chemotherapy RPLND at the urology department of Mashhad University of Medical Sciences, from 2016 to 2019 were consecutively included. All patients gave their informed consent before entering the study. Totally 9 patients with nonseminoma germ cell tumors had indications for RPLND in the post chemotherapy setting, based on the CT imaging and serum tumor markers. All cases had the history of radical orchiectomy and chemo-therapy. In all the included patients, residual retroperitoneal masses ≥1 cm were present following systemic chemotherapy sessions and post chemotherapy serum tumor markers were at normal level, including Beta-HCG <3 ng/ml and Alpha-FP <10 ng/ml (24).

 This study was approved by the ethics committee of Mashhad University of Medical Sciences under the number of 940295. All patients gave an informed consent before entering the study.

***Radiotracer injection***

**Figure 1:**
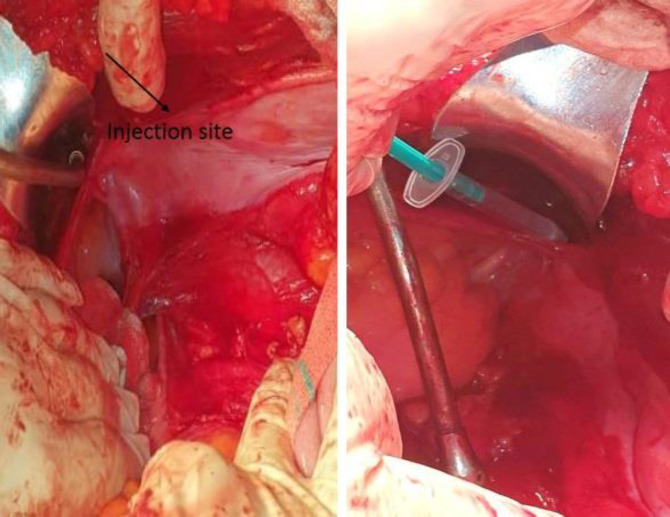
Spermatic cord stump as the injection site of the radiotracer

 Under general anesthesia in the supine position and after exposing the peritoneum through transabdominal approach, 37 MBq (1 mCi) of technetium-99m-labelled phytate in 1 mL of saline was injected in two divided doses (0.5 mL each) in the stump of the spermatic cord (Figure 1).

***Intraoperative detection of sentinel lymph nodes***

 Following exposing the retroperitoneal space, ureter, testicular vein and main vessels were observed. A hand-held gamma probe (SURGEOGUIDE II, Parto Negar Persia Co.), was used for radio-guided retroperitoneal sentinel lymph node detection and confirming the location of the sentinel lymph nodes. The mean time between the injection of the radiotracer to measuring the radioactivity of the lymph nodes was 1.5 hours. Any node with the in vivo count rate, 10 times higher than the thigh count rate was considered as a potential sentinel node and was harvested. Any harvested node with ex vivo counts (30 seconds cumulative count) five times higher than the background (thigh) was considered as a true sentinel lymph node. Ex vivo count was measured by putting the nodes on the tip of the gamma probe and pointing the probe to the ceiling.

 This process was repeated until the instan-taneous count rate of the retroperitoneum became less than 10 times of the background.

***Lymphadenectomy***

 In all patients full bilateral RPLND was performed (24). All the residual masses with the boundaries of the renal hilar vessels (superiorly), ureters (laterally), and common iliac arteries (inferiorly) as the dissection template, were dissected. The lymph node dissection spanned from renal hilar vessels to iliac vessels.

***Pathologic evaluation***

 The pathologists examined the excised sentinel and regional lymph nodes to assess the precise pathological status of retroperitoneal lymph nodes. All dissected tissues were formalin-fixed, paraffin-embedded, and serially sectioned for hematoxylin-eosin staining. The histopatho-logical status of the sentinel nodes was compared with the other dissected lymph nodes.

***Detection rate and false negative rate***

 The detection rate was measured as the ratio of all cases with at least one detected sentinel node to all included patients. The false-negative rate was measured as the ratio of patients with involved non-sentinel lymph node despite pathologically negative sentinel lymph node to all patients with involved nodes and at least one harvested sentinel node (21, 25, 26).

## Results

 Totally, nine candidates for PC-RPLND were included for intraoperative sentinel lymph node mapping in our study. The age median was 29 (range: 17-36) years old. Table 1 shows the demographic data of the patients. 

 In six out of nine patients at least one sentinel lymph node was detected using intraoperative gamma probe, and the detection rate was 66%. In 2/6 patients with successful detection of sentinel node, the pathological evaluation of the dissected lymph nodes showed the metastatic involvement in both sentinel lymph nodes and other resected peritoneal lymph nodes. In 4/6 patients, the dissected sentinel lymph node and other peritoneal lymph nodes were all free of tumoral cells (no false negative case).

**Table 1:** The characteristics of the included patients

**Patients**	**Age**	**BHCG**	**Alpha FP**	**Orchiectomy**	**Detected sentinel lymph node site**	**Number of dissected sentinel lymph nodes**	**Pathological involvement of the sentinel lymph node**	**Pathological status of regional lymph nodes**
1	33	1.6	2.7	Right	Interaortocaval	2	+	+ (1 Interaortocaval)
								
2	36	0.1	4.8	Right	Interaortocaval	2	+	+ (1 Interaortocaval and 1 paraaort))
3	28	1	3.3	Right	NF	-	NF	+
4	29	0.12	2.4	Right	NF	-	NF	+
5	20	1	2	Right	NF	-	NF	+
6	17	2.66	3.89	Right	Internal iliac	1	-	-
7	32	1.2	3.7	Right	External iliac	2	-	-

NF: Not Found, BHCG: Beta-human chorionic gonadotropin, Alpha FP: Alpha-fetoprotein

 Totally, five out of nine cases were positive for tumoral involvement of regional lymph nodes following pathological evaluation; in two of them(patients 1 and 2) the sentinel lymph node was successfully detected and confirmed the observed metastases in regional lymph nodes, and in three of them (patients 3-5) no sentinel lymph node was detected.

**Figure 2:**
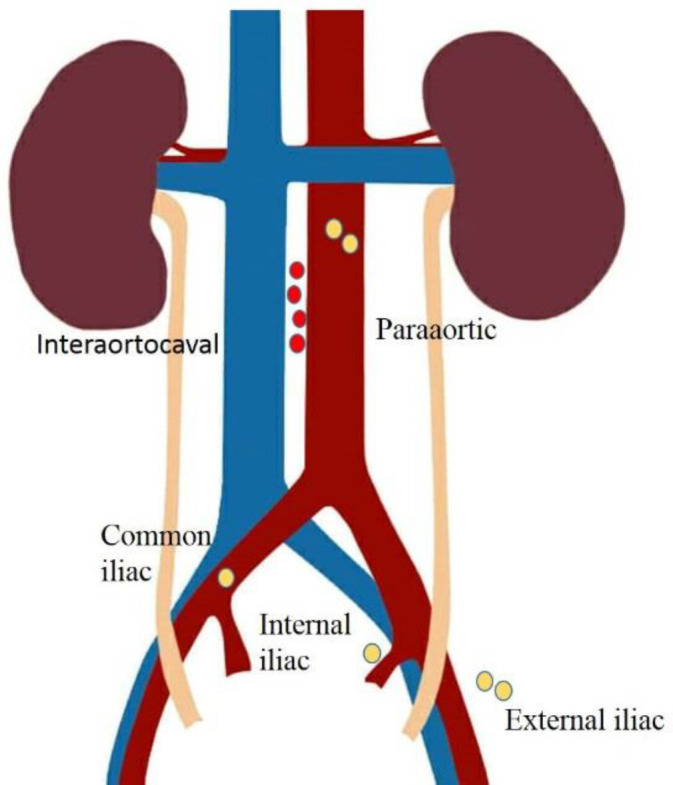
Location of the detected sentinel lymph nodes. Red dots are the metastatic sentinel nodes and yellow dots are non-metastatic sentinel nodes

 Location of the dissected sentinel lymph nodes were interaortocaval (2 patients), internal iliac (1 patient), external iliac (1 patient), common iliac (2 patients), and paraaortic (1 patient), which are indicated in Figure 2.

## Discussion

 In the current study, we evaluated the feasibility of sentinel node biopsy technique in post chemotherapy non-seminomatous testicular cancer patients who were candidate for RPLND. Due to rarity of the cases candidate for post-chemotherapy RPLND, only 9 patients with nonseminoma GCT could be included in our study.

 Thus far, only two other groups reported the feasibility of sentinel node mapping in testicular cancers (23, 27). Brief results of these two groups can be found in Table 2.

**Table 2:** Review of the previous studies

**Studies**	**Country**	**Year**	**Patients**	**Technique**	**Injection**	**Detection rate**	**Patients with metastatic sentinel lymph nodes**	**False negative rate**
Satoh M	Japan	2006	22 with stage I	Gamma probe guided laparoscopic RPLND	15 MBq of ^99m^Tc-nanocolloid phylate in 0.4 ml of saline was injected around the tumor inside the testi-cular tunica albuginea with a 29-gauge butt- erfly needle	21/22 (95%)	3	2/5
Blok JM	Netherlands	2019	27 with stage I	Scintigraphy and/or SPECT/CT Laparoscopic gamma probe	78.9 MBq of ^99m^Tc-nanocolloid in a volume of 0.10–0.20 mL was injected to the testicular parenchyma	25/27	3	0/3
Current study	Iran	…	9	Intraoperative portable gamma probe	37 MBq of technetium-99m-labelled phytate in 1 mL of saline was injected in two divided doses (0.5 mL each) in the stump of the spermatic cord	6/9	2	0/2

 In current study, at least one sentinel node could be harvested intraoperatively in 6 patients (amounts to 66% detection rate). In three out of nine included patients (with the disease stage of IIA-C) we had sentinel lymph node detection failure (33%); these three patients showed the presence of mature teratoma of germ cell origin in retroperitoneal lymph nodes, in pathological report. Several reasons have been proposed for sentinel lymph node detection failure including the methodological variations such as injection technique, and the obstruction of the lymphatic flow due to tumoral involvement of lymph nodes which inhibit the uptake of mapping tracer (22). The main reason for unidentified sentinel lymph nodes in our study was the presence of tumor cells in lymph nodes, which led to the obstruction of the lymphatic flow and inhibition of the radiotracer uptake in lymph nodes, and eventually no radioisotope count could be identified by gamma probe.

 Two previous investigating groups reported the feasibility of preoperative lymphoscinti-graphy and gamma probe guided laparoscopic RPLND in localizing and dissecting the sentinel nodes in patients with seminoma and nonseminoma testicular cancer candidate for orchiectomy. They injected the radiotracer into the testicular tissue around the tumor inside the testicular tunica albuginea one day before the surgery (23, 27). Unlike those studies, our study was performed in post-chemotherapy setting for candidates of full bilateral RPLND. The radiotracer was injected in stump of the spermatic cord at the day of surgery. The history of chemotherapy sessions was another reason of lower detection rate in our study as compared to the other reports. It has been also reported that previous chemotherapy could decrease the success of sentinel node mapping in breast, esophageal, cervical and bladder cancers (22, 28, 29).

 Intraoperative injection of the mapping material has been proven to be a feasible method for sentinel node mapping, as the movement of the tracer in the lymphatics is fairly rapid and successful sentinel node harvesting can be achieved by allowing a reasonable time between injection and harvesting of the sentinel nodes (30-32). Due to reported potential complications of blue dyes, we didn’t use the blue dyes in our study (20, 33).

 False negative rate is the main indicator of the sentinel lymph node mapping diagnostic value. In two out of five cases with metastatic retroperitoneal lymph nodes, sentinel node could be detected successfully which also confirmed the metastatic status of the lymph nodes (pathologically involved sentinel node in 2 cases). In three other patients with the presence of teratoma in dissected peritoneal lymph nodes, sentinel nodes could not be detected intraoperatively. Our results showed a false negative rate of 0% (0/2), which is a promising result. The study of Blok et al. also revealed the presence of micrometastases in three out of 25 evaluated patients through intraoperative gama probe-guided, extraperitoneal laparoscopic-RPLND; they also did not have any false negative detected sentinel lymph node with no nodal recurrence during the follow up (27). In the study of Satoh et al. three cases with stage I testicular cancer revealed micrometastasis in detected sentinel lymph nodes via gamma probe-guided extraperitoneal laparoscopic RPLND, and reported the incidence of nodal relapse in two cases with stage I seminoma (false negative rate of 40% (2/5)) (23). Satoh et al attributed the false negative cases to suboptimal technique of their study. They proposed that standardizing the sentinel lymph node detecting technique can reduce the incidence of false negative rate. To our knowledge, our study is the first on the feasibility of the sentinel node biopsy in patients with nonseminoma GCT who are candidate for RPLND in post-chemotherapy setting. 

## Conclusion

 The sentinel lymph node mapping technique seems to be feasible and promising in post chemotherapy non-seminoma testis cancer patient who are candidate for RPLND; however, further larger studies are needed to increase and standardize the detection rate.

## Conflict of Interest

 The authors declare that they have no conflict of interest.

## Source of Funding

 This study was supported by vice chancellery of research of Mashhad University of Medical Sciences with the approval number of 940295.
